# The emergence of novel infectious diseases and the public health impact of mass gathering events: risks and challenges

**DOI:** 10.3126/nje.v12i4.50997

**Published:** 2022-12-31

**Authors:** Mohammad Asim, Naushad Ahmad Khan, Kudaibergen Osmonaliev, Brijesh Sathian

**Affiliations:** 1 Clinical Research, Trauma and Vascular Surgery, Department of Surgery, Hamad General Hospital, Doha, Qatar; 2 Faculty of Medical Sciences, Ala-too International University, Bishkek, Kyrgyz Republic; 3 Geriatrics and long term care department, Rumailah Hospital, Hamad Medical Corporation, Doha, Qatar; 4 Centre for Midwifery, Maternal and Perinatal Health, Bournemouth University, Bournemouth, UK

## Background

### An overview of emerging infectious diseases

To date, there is the continuous emergence of novel infectious diseases endangering individuals’ health and well-being worldwide. Over the past few decades, around forty contagious diseases, such as Severe Acute Respiratory Syndrome coronavirus-1 (SARS), Middle East Respiratory Syndrome coronavirus (MERS), Ebola, Zika, and newly, COVID-19 and Monkeypox outbreaks, have been reported globally [1]. Zoonotic transmission is the major cause of these emerging infections in humans [2]. These, whether caused by previously unknown pathogens or by those already well-known to science, heighten global concerns about the spread of communicable diseases and the resulting increase in death and disability. [3].

Most importantly, the unprecedented circumstances caused by the Coronavirus disease 2019 (COVID-19) pandemic have greatly impacted the health and socio-economic activities in more than 200 nations. Additionally, this year is remarkable because the COVID-19 pandemic is still ongoing, and to make matters worse, the threat of other serious viral infections, such as Marburg virus and monkeypox, known to spread from human to human, has also recently emerged.

During this pandemic, several intrinsic and extrinsic factors have played crucial roles in influencing disease severity and outcomes. Fortunately, the multidisciplinary effort of researchers worldwide made it possible to understand the pathophysiology of SARS-CoV-2 quickly and successfully developed a COVID-19 vaccine [4]. Nonetheless, the SARS-CoV-2 virus continues to evolve with newer strains. Despite high vaccination rates, data shows that COVID-19 is on the upswing in several countries, and the pandemic appears to be far from over. The recent Omicron variants of the SARS-CoV-2 were reported to be more contagious than the previously circulating Delta variant [5]. As the Omicron wave propagated around the globe, it emerged as the dominant variant in most countries. Therefore, the recent Omicron sub-lineage probably contributes to the recent rise in COVID-19 instances in many continents, including China [6].

Human Monkeypox virus (MPVX) is another zoonotic disease of concern for global public health, which was initially endemic to the west or central Africa and later, in early 2022, spread to many non-endemic countries [7]. Direct contact with the body fluids of infected rodents or primates is a plausible mode of transmission to humans. Also, human-to-human transmission is possible through close direct or indirect contact with an infected person via respiratory droplets [8]. Till October 2022, the World Health Organization (WHO) have reported more than 73,000 cases, including 29 fatalities from different parts of the world [9]. The WHO has designated the growing worldwide epidemic of MPVX as a Public Health Emergency of International Concern due to the rapid increase in reported cases.

Therefore, It is also worth pinpointing that infectious diseases and their accompanying epidemics, as mentioned above, may pose a serious health risk, particularly when occurring concurrently (syndemics) in a country hosting mass gathering event. This is especially true for viral infections, which are more difficult to control than bacterial infectious agents. If proper measures are not followed, these illnesses might spread out of control, triggering epidemics in the host country and other countries after the tourists return to their home countries. Therefore, it is the priority of the host nation to safeguard its population and forestall any worldwide health crisis that may arise due to a mega event like recently concluded Federation Internationale de Football Association (FIFA) World Cup 2022. A diverse public health approach is warranted to control disease transmission in preparing for the health emergencies associated with religious or sport-related mass gathering events.

### Recent mass gathering events and risk of infectious diseases

Public health is at greater risk, as the COVID-19 pandemic is still surging in parts of the world, and also the MPVX is rising, and Marburg disease is re-emerging as a new public health emergency. Mass gathering events present significant public health issues to governments and health agencies during an infectious disease outbreak [10]. To address potential health effects during large-scale gatherings, a new science called mass gathering medicine has evolved recently. Such large-scale events might significantly negatively influence public health, especially during this pandemic [11]. Of note, the emergence of the SARS (2002) and MERS (2012) did not create outbreaks during any mass gathering events across the world [12]. Despite that, the epidemic potential associated with larger gatherings, such as sports and religious, festive, and celebratory events, persists [13]. Initially, in 2020, the COVID-19 pandemic constrained all religious or sports-related activities. The preliminary research indicates that large gatherings, particularly sporting events, may have contributed significantly to the transmission of COVID-19 in several countries, particularly in the early phase of the pandemic [14]. However, these mass gathering events gradually recommenced with implementing preventive measures, control of infection rate and mass rollout of vaccination programs [15].

In the most recent epidemic, several MPVX infections were linked to events such as festivals and situations that promoted close contact [16]. In 2022, the COVID-19 pandemic restrictions were lifted after two and a half years, with Makkah hosted Hajj as the largest mass gathering event during the pandemic, with an attendance of one million pilgrims worldwide. Interestingly, there was no report of MPVX cases among pilgrims who attended Hajj that year [11,17]. Also, the mitigation measures for COVID-19, such as participation of Hajj pilgrims younger than 65, complete COVID-19 vaccination, and pre-travel negative PCR test, lead to a safe and successful event.

Recently, Qatar raised the banner for one of the world’s most prestigious mega-sporting events FIFA World Cup 2022 (November 20th to December 18th, 2022). During this time, the country welcomed over 1.4 million tourists coming from every continent, raising the possibility of a substantial threat to public health [18]. This event was more challenging due to the huge crowd, unlike Tokyo Olympics 2020 (2021), which was hosted without visiting spectators. Notably, before the FIFA World Cup 2022, there were only three confirmed cases of MPVX in Qatar until September 2022. However, it was speculated that the mass gathering might become an epicentre of viral transmission, particularly MPVX [4]. The FIFA World Cup was unquestionably a watershed moment in sports history. However, given the present global context, the public health system of the country hosting the event must be prepared to avoid the transmission of zoonotic diseases as such mega events might considerably enhance the potential of a surge in the number of cases of COVID-19, monkeypox and uncommon Marburg virus sickness, spread throughout the host nation. Therefore, to protect this large gathering from potential health risks or public health issues, the State of Qatar and the WHO have teamed together.

No viral outbreaks have been reported during and after the post-FIFA world cup 2022. This could be attributed to Qatar’s previous experience of holding several international football tournaments with and without fans during the COVID-19 pandemic successfully, as well as adequately tested healthcare management strategies to minimize the risks of infection among participants and spectators [19-22]. The public health initiatives, which were previously successful during the COVID-19 pandemic and included geo-localizing infected individuals, lockdown, medical centre management, and precise re-opening of international borders, undoubtedly assisted Qatar in hosting a successful World Cup that was infection-free.

Overall, the ongoing surge in SARS-Cov-2 cases casts a shadow on the COVID-19 pandemic’s potential course. Omicron may appear anecdotally to be milder than Delta or other variations of concern. Still, when the virus mutates, the global exponential rise of severely mutated Omicron variants will probably result in more fatal strains. In addition, it is critical to assess the recent transmission of monkeypox in light of the continuing COVID-19 pandemic and the possibility of SARS-CoV-2 coinfection with the monkeypox virus. This might lead to variations in the pattern of infection, severity, treatment, or vaccine response in one or both diseases [17].

Nevertheless, the numerous lessons we have learnt about effectively defending ourselves against the spread of viral infections could be attributed to the COVID-19. Naturally, not all illnesses would react to treatments as they did for COVID-19. In this regard, Qatar’s successful hosting of the FIFA World Cup 2022 has been exemplary. It has given hope and a way forward for other nations to follow public health measures to be implemented for hosting f mass gathering events amidst future pandemics.

## Conclusion

To conclude, the COVID-19 pandemic has provided a wealth of knowledge for lowering the risk of infectious diseases. Many preventive measures that were effective for COVID-19 would likely be helpful for other infectious diseases, although not always. An continuing challenge is the emergence of novel and high pathogenic potential that make the sudden outbreak of infectious diseases unpredictable. The ongoing COVID-19 pandemic, with the appearance of novel variations and the potential of vaccine escape, and the co-occurance of a pandemic of monkeypox dominate the global concern about the infectious disease risks linked with the FIFA World Cup 2022 this year in Qatar. The nature of these infectious disease threat necessitates continuous monitoring and surveillance. The development and adaption of efficient mitigation techniques for large gatherings required successful approaches for combating emergent infectious diseases and comparison of public health responses that are benchmarked from earlier successful events during the COVID-19 pandemic.
